# Chagas Disease Has Now Gone Global

**DOI:** 10.1371/journal.pntd.0001136

**Published:** 2011-04-26

**Authors:** Herbert B. Tanowitz, Louis M. Weiss, Susan P. Montgomery

**Affiliations:** 1 Department of Pathology (Division of Parasitology), Albert Einstein College of Medicine, Bronx, New York, United States of America; 2 Department of Medicine (Division of Infectious Disease), Albert Einstein College of Medicine, Bronx, New York, United States of America; 3 Global Health Center, Albert Einstein College of Medicine, Bronx, New York, United States of America; 4 Jacobi Medical Center (Diagnostic Parasitology Laboratory), Bronx, New York, United States of America; 5 Division of Parasitic Diseases and Malaria, Centers for Disease Control and Prevention, Atlanta, Georgia, United States of America; National Institutes of Health, United States of America

Chagas disease, caused by the parasite *Trypanosoma cruzi,* was once thought to be an exotic disease, confined to endemic areas of Latin America and hence of little importance to anyone outside of these endemic regions, including most physicians and scientists. The impact of the lack of physician awareness and lack of scientific attention is undefined, but may contribute to the continued neglect of Chagas disease and the affected populations. Despite historical evidence and growing recognition of the spread of Chagas disease, the prevention and control of this disease outside of Latin America is only now being addressed.

Chagas disease was recognized in the United States as early as the 1950s, when the first reports of local vector-borne cases were published [1]. More recently, immigration patterns from endemic countries have changed the epidemiology of this disease in the US. In 1985, Kirchhoff reported three Bolivian immigrants who presented to the US National Institutes of Health with clinical Chagas disease [2], and in 1987 a survey of Central American immigrants in the Washington, D.C., area revealed a 4.9% prevalence of Chagas disease in this population [3]. Shortly after these reports, cases of transfusion-associated Chagas disease were identified in New York City, US, and Manitoba, Canada [4], [5]. In the New York City case, the donor was traced to a Bolivian immigrant and the recipient was a 12-year-old girl with Hodgkin’s disease. Kirchhoff, in an accompanying editorial, raised the alarm as to whether the blood supply was safe [6]; however, it was not until 15 years later that a screening test for Chagas disease was approved by FDA and implemented by the American blood banking industry. To date, this screening has resulted in the recognition of over 1,300 cases of Chagas disease in donors (http://www.aabb.org/programs/biovigilance/Pages/chagas.aspx), the vast majority of which have been asymptomatic representing the indeterminate form of chronic infection.

In other parts of the world, immigration alone has contributed to the appearance of Chagas disease in non-endemic countries [7]–[10]. Immigration from endemic regions is widespread; for example, there are Brazilian immigrants in Portugal and Bolivian immigrants in Spain, and currently, there are an estimated 100,000 or more Latin American immigrants living in France. With immigration has come Chagas disease. Chagasic heart disease has been reported in Brazilian immigrants of Japanese origin in Japan [8], and the seroprevalence of Chagas disease among Bolivian women in Barcelona has been determined to be 3.4% [8].

In all parts of the world where people at risk for Chagas disease are found, Chagas disease in immune-suppressed patients has become an important consideration, resulting in organ and tissue safety concerns related to both donors and recipients. In non-endemic areas, screening of donors or recipients may not be performed routinely. Furthermore, individuals with chronic Chagas disease who acquire HIV/AIDS may have a recrudescence of the infection that can go unrecognized or misdiagnosed as *Toxoplasma* encephalitis.

Most of those infected have the indeterminate, asymptomatic form of Chagas disease and are unaware of their infection, but remain potential sources of transmission. Pregnant women unaware of their infection can be sources of congenital transmission. Congenital Chagas disease has now been reported in Europe among infants born to mothers who are Latin American immigrants with undiagnosed Chagas disease [11]–[13]. These observations raise the issue as to whether prescreening of pregnant women for Chagas disease should be recommended for immigrants from Chagas-endemic areas. This issue was recently highlighted in a paper by Verani et al. [14], who conducted a survey of obstetricians and gynecologists in the US, and demonstrated that clinicians had an inadequate understanding of basic information about this disease and no knowledge of the fact that Chagas disease could be transmitted from mother to child.

The paper in this issue of *PLoS Neglected Tropical Diseases* by Roca et al. [15] examined the prevalence of Chagas disease among Latin American immigrants in a primary care setting in Barcelona, which has become a destination of Spanish-speaking immigrants from Chagas-endemic areas. Of the 766 patients tested, 22 individuals were diagnosed with *T. cruzi* infection (a prevalence of 2.8%); more women were positive than men (54.6% versus 45.5%). Interestingly, 21 patients were from Bolivia, which is a highly endemic area. The prevalence rate among Bolivian immigrants in this study was 16.5%. Many had lived in substandard adobe houses that have been associated with risk for transmission while in Bolivia, and had previous knowledge of Chagas disease in their country of origin. A number of these patients had clinical Chagas disease, including cardiac and gastrointestinal manifestations.

Awareness that Chagas disease is now found in places far from endemic areas of Latin America is important because it leads to the development of strategies to prevent potential sources of transmission (e.g., blood transfusion, organ transplantation, or congenital transmission), and to identify individuals who may benefit from anti-parasitic therapy. Increased awareness also enables us to identify patients who may have a diagnosis of ischemic heart disease or cardiomyopathy of unknown etiology or individuals with gastrointestinal disorders of unknown etiology whose illness is actually Chagas disease, improving the ability of physicians to care for these patients appropriately. Indeed, it is evident that the challenges of Chagas disease have become global.
